# Assessment of left ventricular systolic and diastolic abnormalities in patients with hypertrophic cardiomyopathy using real-time three-dimensional echocardiography and two-dimensional speckle tracking imaging

**DOI:** 10.1186/s12947-018-0142-y

**Published:** 2018-10-02

**Authors:** Xin Huang, Yan Yue, Yinmeng Wang, Yujiao Deng, Lu Liu, Yanqi Di, Shasha Sun, Deyou Chen, Li Fan, Jian Cao

**Affiliations:** 1 Department of cardiology, Nanlou Division, Chinese PLA General Hospital, National Clinical Research Center for Geriatric Diseases, Beijing, 100853 China; 2 Department of medical administration, Chinese PLA General Hospital, Beijing, 100853 China; 3 Department of Respiration, Clifford Hospital, Guangzhou, 511495 China; 4 Department of Ultrasound, Chinese PLA General Hospital, Beijing, 100853 China; 5 Department of Outpatient, Chinese PLA General Hospital, Beijing, 100853 China

**Keywords:** Hypertrophic cardiomyopathy, Left ventricular, Real-time tri-plane echocardiography and quantitative tissue velocity imaging, Real-time three-dimensional, Two-dimensional speckle tracking imaging, Systolic functions, Diastolic functions

## Abstract

**Background:**

Conventional echocardiography is not sensitive enough to assess left ventricular (LV) dysfunction in hypertrophic cardiomyopathy (HCM) patients. This research attempts to find a new ultrasonic technology to better assess LV diastolic function, systolic function, and myocardial longitudinal and circumferential systolic strain of segments with different thicknesses in HCM patients.

**Methods:**

This study included 50 patients with HCM and 40 healthy subjects as controls. The peak early and late mitral annulus diastolic velocities at six loci (E_a_′ and A_a_′, respectively) and the E_a_′/A_a_′ ratio were measured using real-time tri-plane echocardiography and quantitative tissue velocity imaging (RT-3PE-QTVI). The mean value of E_a_′ at six loci (E_m_′) was obtained for the calculation of E/E_m_′ ratio. The LV end-diastolic volume (LVEDV), LV end-systolic volume (LVESV), LV stroke volume (LVSV), and LV ejection fraction (LVEF) were measured using real-time three-dimensional echocardiography (RT-3DE). LV myocardial longitudinal peak systolic strain (LPSS) and circumferential peak systolic strain (CPSS) in the apical-middle-basal segments (LPSS_-api_, LPSS_-mid_, LPSS_-bas_; CPSS_-api_, CPSS_-mid_, and CPSS_-bas_, respectively) were obtained using a software for two-dimensional speckle tracking imaging (2D-STI). According to the different segmental thicknesses (STs) in each HCM patient, the values (LPSS and CPSS) of all the myocardial segments were categorized into three groups and the respective averages were computed.

**Results:**

The E_a_′, A_a_′, and, E_a_′/A_a_’ ratio in HCM patients were lower than those in the controls (all *p* < 0.001), while the E/E_m_′ ratio in HCM patients was higher than that in the controls (*p* < 0.001). The LVEDV, LVSV, and LVEF were significantly lower in HCM patients than in controls (all *p* < 0.001). In HCM patients, the LPSS_-api_, LPSS_-mid_, LPSS_-bas_, CPSS_-api_, CPSS_-mid,_ and CPSS_-bas_ and the LPSS and CPSS of LV segments with different thicknesses were all significantly reduced (all *p* < 0.001).

**Conclusions:**

In HCM patients, myocardial dysfunction was widespread not only in the obviously hypertrophic segments but also in the non-hypertrophic segments; the LV systolic and diastolic functions were damaged, even with a normal LVEF. LV diastolic dysfunction, systolic dysfunction, and myocardial deformation impairment in HCM patients can be sensitively revealed by RT-3PE-QTVI, RT-3DE, and 2D-STI.

## Background

Hypertrophic cardiomyopathy (HCM) is an autosomal dominant genetic disease caused by a genetic mutation in the myocardial sarcomere gene, and is characterized by myocardial hypertrophy, interstitial fibrosis, myocyte disarray, and asymmetrical left ventricular (LV) hypertrophy [1, 2]. Conventional echocardiography has been used to diagnose diastolic dysfunction, which is characterized by impaired left ventricular relaxation with increased stiffness of the left ventricle and diminished filling rates in HCM patients. However, systolic dysfunction cannot be easily determined using conventional methods. Therefore, we often describe systolic function as normal or hyperdynamic in HCM patients, depending on normal or supernormal LV ejection fraction (LVEF). HCM patients often experience symptoms such as breathlessness, fatigue, and reduced exercise capacity [3]. Previously, it was believed that diastolic dysfunction is the predominant cause of these symptoms. However, molecular studies have confirmed that in HCM, cardiac contractile dysfunction occurs before myocardial hypertrophy [4, 5]. Further, several studies have shown that the contractile force of the cardiac myocytes is impaired, despite the apparently normal or enhanced LVEF observed on conventional echocardiography [6, 7]. Therefore, it is important to evaluate the cardiac function of HCM patients using some novel techniques. We designed this study with the aim of quantifying systolic dysfunction and myocardial deformation impairment in HCM patients using real-time three-dimensional echocardiography (RT-3DE) and two-dimensional speckle tracking imaging (2D-STI). Furthermore, LV diastolic function can be evaluated easily and quickly by measuring the mitral valve flow spectrum, but some patients in the pseudonormalization spectrum cannot be clearly assessed by traditional ultrasound technology. Therefore, in this study, measurement of diastolic velocity at six loci in mitral annulus using real-time tri-plane echocardiography and quantitative tissue velocity imaging (3PE-QTVI) was designed for the evaluation of LV diastolic function.

## Methods

### Study population

The study population included 50 patients with non-obstructive HCM, and they were consecutively selected. These patients had undergone clinical and echocardiographic evaluations at our hospital between October 2016 and November 2017. The inclusion criteria [8] were as follows: (1) A 2D ultrasound showing asymmetric hypertrophy in the LV walls; (2) septum/posterior wall ratio ≥ 1.5; and (3) inter-ventricular septum thickness ≥ 1.5 mm. The exclusion criteria were as follows: (1) ventricular hypertrophy caused by simple apical HCM; (2) resting pressure gradient of the LV outflow tract ≥30 mmHg; (3) end-stage HCM (LVEF < 50%); (4) history of invasive septal reduction therapy (septal myomectomy or alcohol septal ablation); (5) organic (degenerative or rheumatic) valvular disease; (6) myocardial ischemia noted during noninvasive investigation, suggesting coronary artery disease; and (7) prior history of myocardial infarction or myocarditis. According to the segmental thickness (ST) in the diastole, all the segments of HCM patients were divided into three groups: HCM-A group (ST < 12 mm; 227 segments), HCM-B group (ST, 12–15 mm; 281 segments), and HCM-C group (ST > 15 mm; 392 segments). For the healthy control group, 40 age- and gender-matched participants were selected from the same time frame based on the following conditions: (1) normal results on physical examination, echocardiography, electrocardiography (ECG), and biochemistry tests and (2) no coronary artery disease, hypertension, valvular disease, arrhythmia, and systemic disease.

### Prepared apparatus

Conventional echocardiography and 2D-STI were performed in all participants, using the Vivid 7-dimension ultrasound machine and an M3S probe. The 3PE-QTVI and RT-3DE examinations were performed using a 3 V probe.

### Standard ultrasound examination

The LV end-diastolic diameter (LVDd), LV end-systolic diameter (LVDs), LV posterior wall thickness (LVPWT), inter-ventricular septal thickness (IVST), and left atrial end-systolic dimension (LADs) were measured along the LV long-axis view. The IVST/LVPWT ratio was calculated. The pulsed wave sampling volume was placed under the tips of the mitral valve leaflets from the apical four-chamber view. The peak early and late mitral inflow diastolic velocities (E and A, respectively) were measured using pulse Doppler, and the E/A ratio was calculated.

### 3PE-QTVI examination

During 3PE-QTVI, LV apical (long-axis and four- and two-chamber) views were obtained synchronously in cardiac cycles. The sampling volume was placed at six loci in the mitral annulus, namely, anterior septum, anterior wall, anterolateral wall, inferolateral wall, inferior wall, and inferior septum. The peak early and late mitral annulus diastolic velocity (E_a_′ and A_a_′, respectively) and E_a_′/A_a_’ ratio were measured by 3PE-QTVI. The mean value of E_a_′ at the six loci (E_m_′) was obtained for the calculation of E/E_m_′.

### RT-3DE examination

RT-3DE full volume images were acquired during an end-expiratory breath-hold using a wide-angle acquisition mode in which four wedge-shaped subvolumes were obtained from four consecutive cardiac cycles with the acquisition triggered to the R wave of the ECG; subsequently, these datasets were stored and transferred to a computer for offline analysis. LV end-diastolic volume (LVEDV), LV end-systolic volume (LVESV), LV stroke volume (LVSV), and LVEF were calculated using a four-dimensional LV analysis software.

### 2D-STI examination

High frame rate images of the LV long-axis, four- and two-chamber, and short-axis views of the basal, middle, and apical segments were obtained, respectively. Three consecutive cardiac cycles were acquired in each view and saved in a cine-loop format for offline analysis supported by a digitized software package. The software package automatically tracked the changes in the width of the region of interest, which was adjusted, as required, to fit the wall thickness. The automatic algorithm obtained an 18-segment model and provided the longitudinal peak systolic strain (LPSS) and circumferential peak systolic strain (CPSS) for each segment. Subsequently, the average LPSS and CPSS values of basal, middle, and apical segments (LPSS_-bas_, LPSS_-mid_, LPSS_-api_; CPSS_-bas_, CPSS_-mid_, and CPSS_-api_, respectively) were calculated. The values (LPSS and CPSS) of all the myocardial segments for each HCM patient were categorized into three groups (HCM-A, HCM-B, and HCM-C) according to the ST, and subsequently, the average was computed.

The aforementioned ultrasound parameters were measured according to the American Society of Echocardiography guidelines and requirements [9].

### Statistical analysis

Data analysis was performed using the Statistical Package for the Social Sciences software (SPSS, Version 19.0). The results are presented as mean ± standard deviation. The normality test was used to compare continuous variables, and the one-way analysis of variance was used to compare the echocardiographic values in HCM patients and the control group. Continuous variables obtained from different groups were compared using the Fisher’s least significant difference test. All the *p* values were two-sided, and *p* < 0.05 was considered statistically significant.

## Results

### General information

The clinical characteristics of HCM patients and the control group are shown in Table 1. Both the groups were well matched with respect to age and gender. No statistical differences were observed in the body surface area, heart rate, and blood pressure between the two groups (all *p* > 0.05).

**Table 1:** Clinical characteristics and conventional echocardiographic parameters in HCM patients versus control group

	HCM patients (*N* = 50)	Control group (*N* = 40)	*p* value
Age(years)	46.67 ± 11.09	43.93 ± 8.17	0.195
Gender			
Male	30 (60%)	24 (60%)	
Female	20 (40%)	16 (40%)	
HR(beats/min)	70.51 ± 9.62	68.43 ± 11.95	0.362
BSA(m^2^)	1.72 ± 0.15	1.70 ± 0.21	0.612
SBP(mmHg)	124.63 ± 13.05	120.59 ± 9.67	0.106
DBP(mmHg)	78.32 ± 11.57	74.81 ± 9.07	0.119
IVST (mm)	25.33 ± 7.11	9.32 ± 1.35	< 0.001
LVPWT (mm)	14.25 ± 3.15	8.84 ± 1.11	< 0.001
septum/posterior wall ratio	1.86 ± 0.69	1.05 ± 0.10	< 0.001
LVDd (mm)	40.83 ± 4.87	44.16 ± 2.44	< 0.001
LVDs (mm)	23.32 ± 4.09	27.81 ± 1.64	< 0.001
LADs	40.91 ± 0.83	31.85 ± 1.95	< 0.001
E (cm/sec)	79.04 ± 3.26	80.36 ± 3.07	0.053
A (cm/sec)	71.05 ± 2.89	69.89 ± 2.65	0.052
E /A ratio	1.12 ± 0.39	1.15 ± 0.29	0.675

*HR* heart rate, *BSA* body surface area, *SBP* systolic blood pressure, *DBP* diastolic blood pressure, *IVST* inter-ventricular septal thickness, *LVDd* left ventricular end-diastolic diameter, *LVDs* left ventricular end-systolic diameter, *LVPWT* left ventricular posterior wall thickness, *LADs* left atrium end-systolic dimension. E: mitral inflow peak velocity in early diastole; A: mitral inflow peak velocity in late diastole. Values are expressed as the mean ± SD

### Conventional echocardiographic parameters

The IVST, LVPWT, septum/posterior wall ratio, and LADs were significantly higher in HCM patients than in the control group (all *p* < 0.001), whereas LVDd and LVDs were significantly lower in HCM patients than in the control group (all *p* < 0.001). Transmitral Doppler measurements showed no significant difference between the peak E, peak A, and E/A in HCM patients and those in the control group (all *p* > 0.05) (Table 1).

### 3PE-QTVI parameters

**Fig 1.**
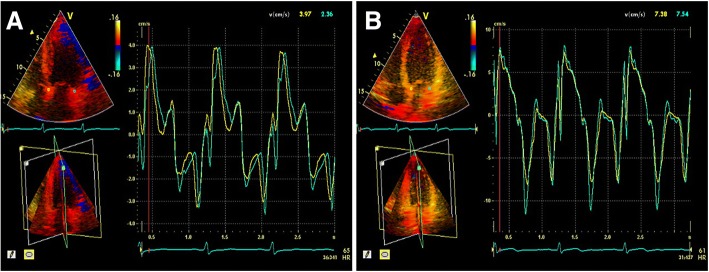
The peak early and late mitral annulus diastolic velocities (Ea’ and Aa’, respectively) at apical four-chamber view were measured using real-time tri-plane echocardiography and quantitative tissue velocity imaging. Ea’ and Aa’ are significantly lower in HCM patients than in control group

In this study, E_a_′, A_a_′, and E_a_′/A_a_′ ratio at the six loci in the mitral annulus in HCM patients were lower than those in the control group, whereas the E/E_m_′ ratio in HCM patients was higher than that in the control group; all the differences were statistically significant (all *p* < 0.001) (Table 2; Fig. 1).

**Table 2:** RT-3PE-QTVI parameters in HCM patients versus control group

	HCM patients (*N* = 50)	Control group (*N* = 40)	*p* value
E_a_^’^(cm/sec)			
AS	3.35 ± 1.15	8.14 ± 2.23	< 0.001
AN	3.58 ± 1.25	8.87 ± 1.81	< 0.001
AL	4.24 ± 1.50	9.25 ± 1.80	< 0.001
IL	3.88 ± 1.44	10.26 ± 2.01	< 0.001
IN	3.51 ± 1.46	9.18 ± 1.57	< 0.001
IS	3.44 ± 1.42	8.20 ± 1.31	< 0.001
A_a_^’^ (cm/sec)			
AS	5.12 ± 1.84	6.48 ± 0.87	< 0.001
AN	5.22 ± 1.20	7.08 ± 0.56	< 0.001
AL	5.93 ± 1.43	7.21 ± 0.65	< 0.001
IL	6.02 ± 1.58	7.59 ± 1.13	< 0.001
IN	5.68 ± 1.46	7.80 ± 1.32	< 0.001
IS	5.10 ± 1.27	6.63 ± 0.68	< 0.001
E _a_^’^ / A_a_^’^			
AS	0.71 ± 0.26	1.27 ± 0.43	< 0.001
AN	0.69 ± 0.20	1.24 ± 0.18	< 0.001
AL	0.74 ± 0.28	1.29 ± 0.23	< 0.001
IL	0.63 ± 0.22	1.45 ± 0.33	< 0.001
IN	0.63 ± 0.25	1.21 ± 0.29	< 0.001
IS	0.69 ± 0.18	1.26 ± 0.32	< 0.001
E/E_m_^’^ ratio	21.47 ± 5.36	10.01 ± 3.94	< 0.001

*E*_*a*_^*’*^ early diastolic velocity of mitral annulus, *A*_*a*_^*’*^ late-diastolic velocity of mitral annulus, *E* mitral inflow peak velocity in early diastole, *E*_*m*_^*’*^ mean value of E_a_^’^ at six loci in mitral annulus, *AS* anterior septum, *AN* anterior wall, *AL* anterolateral wall, *IL* inferolateral wall, *IN* inferior wall, *IS* inferior septum. Values are expressed as the mean ± SD

### RT-3DE parameters

**Fig 2.**
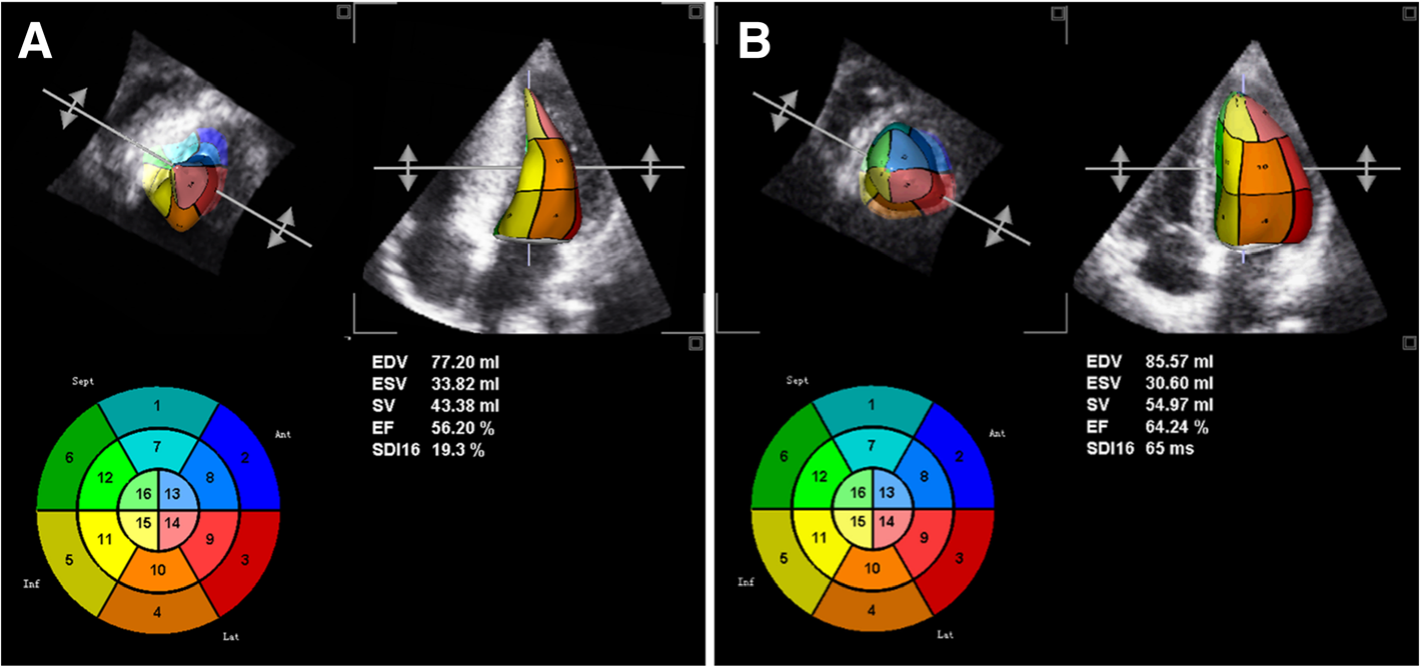
Left ventricular (LV) global shell maps by real-time three-dimensional echocardiography full volume image are shown. Left ventricle is seen as a “wedge-shape” in HCM patients. LV end-diastolic volume (LVEDV), end-systolic volume (LVESV), ejection fraction (LVEF) were significantly lower in HCM patients than in control group

RT-3DE showed that LVEDV, LVSV, and LVEF were significantly lower in HCM patients than in the control group (all *p* < 0.001), although LVEF was within the normal range (LVEF > 50%) (Table 3; Fig. 2).

**Table 3:** RT-3DE parameters in HCM patients versus control group

	HCM patients (N = 50)	Control group (N = 40)	*p* value
LVEDV(ml)	70.12 ± 13.91	85.59 ± 14.94	< 0.001
LVESV (ml)	31.09 ± 6.02	29.46 ± 5.47	0.187
LVSV (ml)	39.94 ± 10.67	56.25 ± 11.68	< 0.001
LVEF(%)	57.17 ± 4.15	65.13 ± 2.95	< 0.001

*LVEDV* left ventricular end-diastolic volume, *LVESV* left ventricular end-systolic volume, *LVSV* left ventricular stroke volume, *LVEF* left ventricular ejection fraction. Values are expressed as the mean ± SD

### 2D-STI parameters

**Fig 3.**
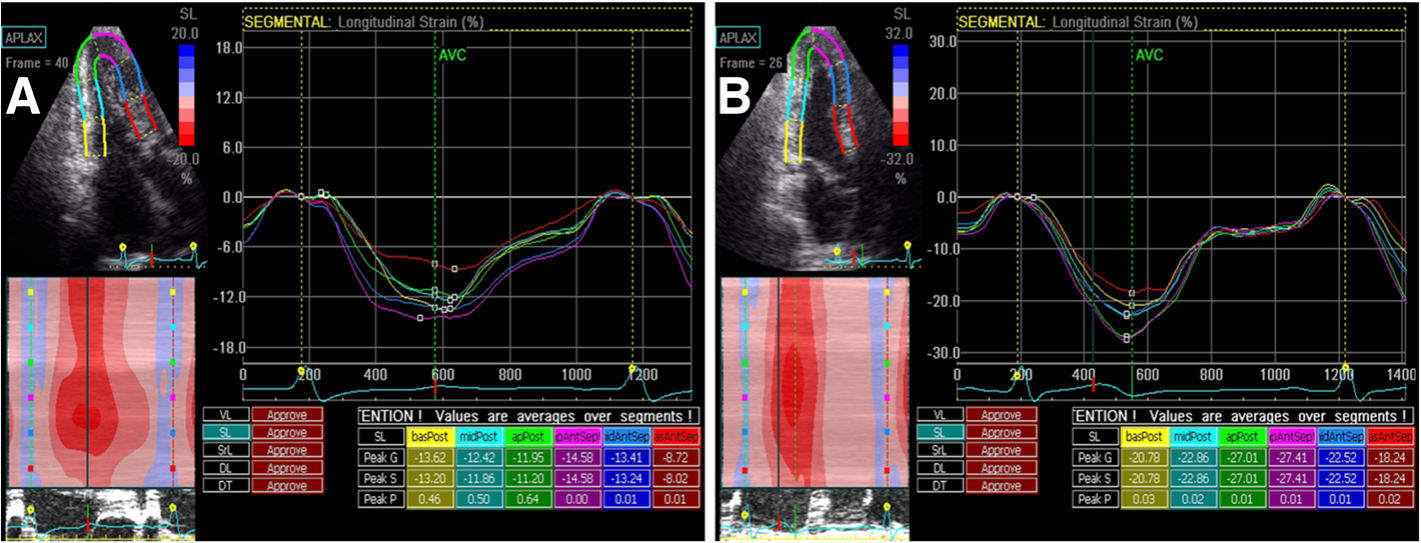
Two-dimensional speckle tracking images in left ventricular apical long-axis view are shown. Left ventricular longitudinal peak systolic strain in HCM patients is significantly lower than in control group

**Fig 4.**
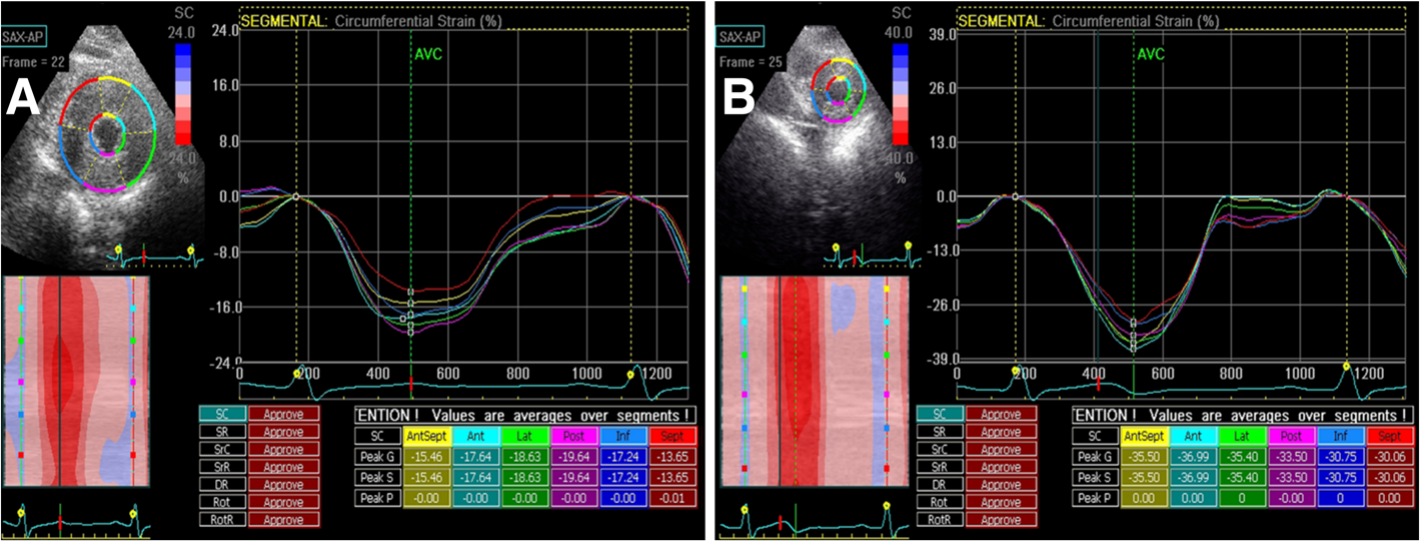
Two-dimensional speckle tracking images in left ventricular short-axis view at the apical level are shown. Left ventricular circumferential peak systolic strain is significantly lower in HCM patients than in control group

A gradual increase in the amplitudes of LPSS and CPSS was observed from the basal segment to the apex in the control group. However, in HCM patients, this regular pattern was not evident, and LPSS_-bas_, LPSS_-mid_, LPSS_-api_, CPSS_-bas_, CPSS_-mid_, and CPSS_-api_ decreased significantly (all *p* < 0.001) (Table 4; Figs. 3 and 4). LPSS and CPSS of the LV myocardial segments in HCM-A, HCM-B, and HCM-C groups were all reduced significantly compared with those in the control group (all *p* < 0.001), and there was a statistically significant difference between LPSS and CPSS of the LV myocardial segments among HCM-A, HCM-B, and HCM-C groups (all *p* < 0.001); a decreased amount of strain correlated with the myocardial thickness grade (Table 5). There was a statistically significant difference in the thickness of the myocardium among HCM-B, HCM-C groups and the control group (all *p* < 0.001); there was no significant difference in the thickness of the myocardium between HCM-A and the control group.

**Table 4:** STI results of longitudinal and circumferential strain in HCM patients versus control group

	HCM patients (N = 50)	Control group (N = 40)	*p* value
LPSS_-bas_	−13.91 ± 5.17	−21.67 ± 3.50	< 0.001
LPSS_-mid_	−12.59 ± 5.83	−22.02 ± 3.06	< 0.001
LPSS_-api_	−14.76 ± 6.84	−24.58 ± 3.96	< 0.001
CPSS_-bas_	−16.99 ± 6.19	−22.13 ± 4.86	< 0.001
CPSS_-mid_	−15.85 ± 5.34	−23.45 ± 3.70	< 0.001
CPSS_-api_	−16.24 ± 4.58	−28.32 ± 4.15	< 0.001

*LPSS* longitudinal peak systolic strain, *CPSS* circumferential peak systolic strain. Values are expressed as the mean ± SD

**Table 5:** STI results of the peak systolic longitudinal and circumferential strain in different segmental thickness(ST) groups in HCM patients and control group

	HCM-A (ST<12 mm, 227segments)	HCM-B (ST:12–15 mm, 281segments)	HCM-C (ST>15 mm, 392 segments)	Control group (720 segments)
ST	9.45 ± 1.25	13.39 ± 1.21^*#^	17.53 ± 1.84^*#&^	9.37 ± 1.18
LPSS	−17.79 ± 3.75^*^	− 13.51 ± 4.02^*#^	−10.03 ± 3.17^*#&^	−22.89 ± 3.41
CPSS	−19.83 ± 4.07^*^	− 16.19 ± 5.71^*#^	−13.32 ± 4.39^*#&^	−24.61 ± 3.52

Values are expressed as the mean ± SD

^*^p < 0.001 vs. Control group’ segment

^#^*p* < 0.001 vs. HCM-A group (ST < 12 mm)

^&^*p* < 0.001 vs. HCM-B group (ST:12 mm − 15 mm)

## Discussion

Owing to the complexity of a three-dimensional cardiac structure, helical ventricular myocardial band (HVMB) is considered to be a critical cornerstone in understanding cardiac function. The HVMB comprises a helical component, namely an apical vortex (oblique descending and ascending segments that cross each other at 60° angles en-route to forming) and a basal loop (containing both left and right transverse sides) [10]. During ventricular contraction, all the components coordinate synergistically to achieve myocardial longitudinal shortening, circumferential narrowing or compression, radial thickening, and twisting in favor of ejection. Because of mutation in the genes encoding the sarcomeric protein of the cardiac contractile apparatus, a series of characteristic pathological changes occur in HCM patients. This study aimed to quantify the effects of the pathological changes on the LV systolic and diastolic functions in HCM patients.

### Evaluation of LV diastolic function in HCM patients by 3PE-QTVI

The Moderate reduction of diastolic function, usually showing E/A ratio > 1, is due a pseudonormalized phenomenon, which is difficult to distinguish from normal cardiac function. In this study, E and A were not significantly different between HCM patients and the control group, and the E/A ratio was > 1, which means that the mitral valve orifice flow spectrum may not truly reflect the changes in the diastolic function in patients with HCM.

The present study reported that the measurement of early and late mitral annulus diastolic velocity can reflect changes in relaxation and compliance of the left ventricle, independent of the cardiac load [3]. In this study, 3PE-QTVI was used for detection. By obtaining three orthogonal views from a 60° angle synchronously, this technology overcomes the limitations of conventional QTVI, such as display of only one view in each cardiac cycle and susceptibility to heart rate and breathing. In this study, E_a_′, A_a_′, and E_a_′/A_a_′ ratio in HCM patients were lower than those in the control group. The results showed that relaxation and compliance of the left ventricle were impaired. In addition, the E/E_m_′ ratio reflects LV end-diastolic pressure (LVEDP). The E/E_m_′ ratio in HCM patients significantly increased compared with that in the control group, indicating that LVEDP was significantly higher in HCM patients. When the myocardium of the left ventricle (main pumping chamber) is of abnormal thickness, the myocardial stiffness increases and compliance decreases, which results in increase in LV filling pressure. Long-term restriction of left atrial to ventricular blood filling may lead to left atrial enlargement. Left atrial dilation negatively affects patient condition and prognosis and may lead to atrial fibrillation, heart failure, sudden death, and other cardiovascular events [11, 12].

### Evaluation of LV pumping function in HCM patients by RT-3DE

RT-3DE can overcomes the limitations of 2D echocardiography by demonstrating a 3D ventricular shape at different time phases and depicting the whole extent of the ventricular endocardium. In this study, LVEDV, LVSV, and LVEF measured by RT-3DE were observed to have significantly reduced in HCM patients compared with those in the control group, although LVEF was in the normal range (LVEF > 50%). Ejection fraction, depending on the percentage of stroke volume in LVEDV, mainly reflects changes in the cardiac cavity dimensions and volume, and is obviously affected by cardiac preload and afterload. The hypertrophic myocardium and papillary muscles protrude into the chamber [13], and reduce LV dimensions and volume, which is seen as a wedge- or spade-shaped ventricular cavity in HCM patients. Therefore, LVEF cannot completely reflect the cardiac stroke volume, especially in HCM patients with obviously decreased LV dimension and volume. Merely applying the parameter to evaluate LV systolic function can cause errors or bias [14, 15]. This study showed that LVSV had significantly decreased, although LVEF was in the normal range (LVEF > 50%). Hence, the LVSV may objectively and sensitively reveal the impairment of LV systolic function in HCM patients, despite a normal LVEF. We have previously demonstrated that symptoms such as syncope, palpitation, breathlessness, and exercise limitation are closely related to diastolic dysfunction. In this study, we considered that those symptoms could also be indicative of reduced LVSV.

### Evaluation of LV myocardial deformation in HCM patients by 2D-STI

In this study, LPSS and CPSS values in apical-middle-basal segments showed a regular pattern in the control group. The amplitudes of LPSS and CPSS increased gradually from the basal segment to the apical segment in the control group. However, this regularity was not evident in HCM patients. The LV LPSS and CPSS values of the basal, middle, and apical segments decreased significantly. This indicated that the LV myocardial systolic function was impaired in HCM patients. This impairment of the LV myocardial systolic function could be caused by the following factors: (1) HCM is characterized by abnormal hypertrophy in the cardiomyocytes, increased collagen, and interstitial fibrosis, and even macroscopically visible myocardial scarring [1, 16]; (2) hypertrophic myocardium and myofibrillar disarray are also considered pathological hallmarks of HCM, these are distributed unevenly and arranged irregularly in the form of swirls or clusters [1, 2, 16]; and (3) microvascular dysfunction occurs in HCM, which features myocardial/vascular ratio imbalance (decrease in myocardial capillary density), coronary artery wall thickening, and development of myocardial ischemia [17, 18].

Furthermore, in this study, LPSS and CPSS of the LV myocardial segments with different thicknesses were reduced significantly in HCM patients, even the normal thickness of the HCM-A group was significantly lower than that of the control group, but there was no significant difference in the thickness of the myocardium between HCM-A and the normal control group. Further, the decreased amount of strain correlated with the myocardial thickness grade. Thus, the myocardial systolic dysfunction not only in the obvious hypertrophic segments but also in the non-hypertrophic segments suggested that myocardial systolic dysfunction in HCM patients may precede myocardial hypertrophy. This result is consistent with the results reported in previous studies [15]. Therefore, myocardial systolic dysfunction may occur first and the hypertrophy could be a compensatory response. The more obvious the magnitude of hypertrophy, the more serious is the impairment of myocardial strain. Just as demonstrated by Urbano-Moral et al. [19], the extent of hypertrophy is the primary factor altering myocardial mechanics. For HCM patients, the degree of myocardial hypertrophy is one of the parameters evaluated during regular follow-ups, and this study suggests that this evaluation may be important for assessing myocardial function, predicting the progress of the disease, and guiding therapy in clinical practice.

### Limitations

Several limitations of this study must be considered. First, because the sample size was rather small, the results obtained from this study may not be statistically strong. Second, evaluation by RT-3DE and 2D-STI was significantly dependent on the quality of image. The assessments were feasible in patients with sufficient images quality. Some situations such as poor acoustic window (resulting in poor images) of cardiac patients with arrhythmia and examiner’s subjective experience could have affected the accuracy and reliability of the data. In this study, the patients with poor images or arrhythmia were ruled out. Third, LVEF of HCM group is significantly lower than that of control group, even though it is within normal range. That can be a basic difference between two groups of this study, and consequently affect the other results comparing two groups. HCM group of this study may be patients with systolic dysfunction as well as diastolic dysfunction. Finally, a bias existed in different echocardiographic software packages from various ultrasound machine vendors, which may have affected the measurement repeatability and accuracy within acceptable limits.

## Conclusions

This study shows that widespread myocardial dysfunction occurred not only in the obvious hypertrophic segments but also in the non-hypertrophic segments. The more obvious the magnitude of hypertrophy, the more serious is the impairment of myocardial strain. The LV systolic and diastolic functions were damaged; however, the LVEF in HCM patients was in the normal range. The LV diastolic dysfunction, systolic dysfunction, and myocardial deformation impairment in HCM patients can be early, objectively, and sensitively revealed by RT-3PE-QTVI, RT-3DE, and 2D-STI.

## Abbreviations

2D-STI: Two-dimensional speckle tracking imaging
3PE-QTVI: Real-time tri-plane echocardiography and quantitative tissue velocity imaging
A: The peak late mitral annulus diastolic velocities
A_a_′: The peak late mitral annulus diastolic velocities at six loci
CPSS: Circumferential peak systolic strain
CPSS_-api_: Circumferential peak systolic strain in the apical segments
CPSS_-bas_: Circumferential peak systolic strain in the basal segments
CPSS_-mid_: Circumferential peak systolic strain in the middle segments
E: The peak early mitral annulus diastolic velocities
E_a_′: The peak early mitral annulus diastolic velocities at six loci
E_m_′: The mean value of E_a_′ at six loci
HCM: Hypertrophic cardiomyopathy
HCM-A: Segmental thickness < 12 mm
HCM-B: Segmental thickness: 12–15 mm
HCM-C: Segmental thickness > 15 mm
HVMB: Helical ventricular myocardial band
IVST: Inter-ventricular septal thickness
LADs: Left atrial end-systolic dimension
LPSS: Longitudinal peak systolic strain
LPSS_-api_: Longitudinal peak systolic strain in the apical segments
LPSS_-bas_: Longitudinal peak systolic strain in the basal segments
LPSS_-mid_: Longitudinal peak systolic strain in the middle segments
LV: Left ventricular
LVDd: Left ventricular end-diastolic diameter
LVDs: Left ventricular end-systolic diameter
LVEDP: Left ventricular end-diastolic pressure
LVEDV: Left ventricular end-diastolic volume
LVEF: Left ventricular ejection fraction
LVESV: Left ventricular end-systolic volume
LVPWT: Left ventricular posterior wall thickness
LVSV: Left ventricular stroke volume
RT-3DE: real-time three-dimensional echocardiography
ST: Segmental thickness
